# Using Integrated Bite Case Management to estimate the burden of rabies and evaluate surveillance in Oriental Mindoro, Philippines

**DOI:** 10.20517/ohir.2023.02

**Published:** 2023-08-31

**Authors:** Catherine Swedberg, Mary Elizabeth G. Miranda, Criselda Bautista, David Anderson, Marife Basa-Tulio, Nai Rui Chng, Van Denn D. Cruz, Mikolaj Kundegorski, Jobin Maestro, Daria Manalo, Klaudyna Maniszewska, Duane R. Manzanilla, Stella Mazeri, Richard J. Mellanby, Sheryl Pablo-Abarquez, Beatriz Quiambao, Shynee Vee M. Telmo, Caroline Trotter, Mirava Yuson, Katie Hampson

**Affiliations:** 1 School of Biodiversity, One Health & Veterinary Medicine, College of Medical, Veterinary & Life Sciences, University of Glasgow, Glasgow G12 8QQ, Scotland, United Kingdom; 2 Field Epidemiology Training Program Alumni Foundation, Inc. (FETPAFI), Quezon City 1101, Metro Manila, Philippines; 3 Research Institute for Tropical Medicine (RITM), Muntinlupa 1781, Metro Manila, Philippines; 4 Provincial Health Office, Calapan 5200, Oriental Mindoro, Philippines; 5 School of Health and Wellbeing, College of Medical, Veterinary & Life Sciences, University of Glasgow, Glasgow G12 8QQ, Scotland, United Kingdom; 6 Municipal Health Office, Alcantara 5500, Romblon, Philippines; 7 Department of Agriculture Regional Animal Disease Diagnostic Laboratory (RADDL), Naujan 5204, Oriental Mindoro, Philippines; 8 Royal (Dick) School of Veterinary Studies and the Roslin Institute, University of Edinburgh, Easter Bush Campus, Midlothian EH25 9RG, Scotland, United Kingdom; 9 Departments of Veterinary Medicine and Pathology, University of Cambridge, Cambridge CB3 0ES, United Kingdom

**Keywords:** Dog-mediated rabies, integrated bite case management, one health, post-exposure prophylaxis, surveillance

## Abstract

**Background:**

Despite national elimination efforts, dog-mediated rabies remains endemic in the Philippines. Free provision of post-exposure prophylaxis (PEP) through the widespread establishment of Animal Bite Treatment Centers (ABTCs) has improved accessibility; however, the resulting upsurge in PEP demand is not sustainable, and human rabies deaths continue. Dog vaccination coverage also remains inadequate, and it is unclear whether surveillance is effective.

**Methods:**

Here, we used Integrated Bite Case Management (IBCM) to collect enhanced rabies surveillance data in Oriental Mindoro Province over a 3-year period (2020-2022). Adapting a probabilistic decision tree model, we estimated the burden of rabies, evaluated surveillance performance, and analyzed the costs and benefits of current rabies prevention and control practices in the province.

**Results:**

The incidence of bite patients receiving PEP was high in Oriental Mindoro Province (1,246/100,000 persons/year), though < 3% of presenting patients were deemed high-risk for rabies exposure (24/100,000 persons/year). Using a decision tree model, we estimated that around 73.8% of probable rabies-exposed patients sought PEP (95% Prediction Interval, PrI: 59.4%-81.1%) and that routine surveillance confirmed < 2% of circulating animal rabies cases, whereas IBCM resulted in a nearly fourfold increase in case detection. Furthermore, we estimated that an average of 560 (95% PrI 217-1,090) dogs may develop rabies annually in the province, equating to 3-5 cases per 1,000 dogs per year. On average, 20 to 43 human deaths were averted by PEP each year in Oriental Mindoro at an annual cost of $582,110 USD (i.e., $51.44 USD per person) or $20,190 USD (95% PrI $11,565-79,400) per death averted.

**Conclusion:**

While current practices for PEP provisioning in the Philippines have improved access, a large proportion of people exposed to rabies (> 26%, 95% PrI 18.8%-40.1%) are still not seeking healthcare. Integrating an intersectoral surveillance system, such as IBCM, into national policy could greatly improve case detection if well implemented, with further benefits extending to guidance for PEP administration, potentially reducing unnecessary expenditure on PEP, and situational awareness to inform control of rabies through mass dog vaccination.

## Introduction

Rabies, a lethal viral zoonotic disease, requires prompt administration of post-exposure prophylaxis (PEP) to prevent the onset of infection^[1]^. Nearly all of the estimated 59,000 annual human rabies deaths are attributed to transmission through bites from domestic dogs in low- and middle-income countries (LMICs) across Africa and Asia^[2,3]^. However, like other neglected diseases, the true burden of rabies remains unknown, with only a fraction of human and animal cases reported in official medical and veterinary records. This assumed underreporting is primarily due to the ineffectiveness and unreliability of passive surveillance systems in endemic regions, resulting in reduced advocacy, funding, and engagement in rabies control initiatives^[4,5]^. Implementation of effective strategies to enhance case detection is therefore imperative for championing, guiding, and evaluating rabies control programs to achieve elimination.

Dog rabies was first confirmed in the Philippines in 1910 when a human case was reported and Negri bodies were identified in the brain of the biting dog^[6]^. Since then, the national government has been leading rabies control efforts aimed at eliminating rabies from the country. In 2007, the National Rabies Prevention and Control Program (NRPCP) was mandated under the Anti-Rabies Act (Republic Act No. 9482), which included the widespread establishment of Animal Bite Treatment Centers (ABTCs) and their free provision of PEP to bite victims^[7]^. However, despite this policy’s success in improving the accessibility of PEP, the number of patients seeking care for dog bites has increased more than fivefold since its introduction (from ~200 to over 1,000 patients per 100,000 people/year)^[8]^. Moreover, dog-mediated rabies remains endemic throughout most of the Philippines and reductions in rabies deaths have plateaued at around 200-300 reported annually. Yet, due to incomplete surveillance data, the true number of deaths is presumably higher.

Integrated Bite Case Management (IBCM) is recommended by WHO and global partners as a gold standard method to strengthen rabies surveillance in LMICs and support the goal of ending human deaths from dog-mediated rabies by the year 2030 (Zero by 30)^[9,10]^. Embracing a One Health approach, IBCM fosters intersectoral collaboration and coordination, acknowledging the interdependence of the health of humans, animals, and their shared environment^[11]^. Previous case studies of IBCM have been implemented in the Philippines on the island of Bohol^[12]^ and in Albay Province^[13]^. IBCM utilizes bite patient risk assessments to identify potential rabies exposures from suspect rabid animals, which are then investigated, with samples collected for laboratory confirmation in dead/euthanized animals. By enhancing surveillance, IBCM has the potential to provide more accurate estimates of rabies burden and to be used for informing the implementation of control and prevention measures, including PEP administration decisions to reduce unnecessary use, and evaluation of the progress/impact of elimination programs, including advocating for increased investment^[14]^.

This study aims to assess the value of integrating an intersectoral surveillance system such as IBCM into national policy to enhance rabies case detection and support the Philippines in achieving rabies freedom by 2030. In this research, we adapted a decision tree framework^[15]^ with the following objectives: (1) to improve the accuracy of estimations for the burden of rabies in the province of Oriental Mindoro; (2) to evaluate the performance of existing surveillance systems; and (3) to analyze the costs and benefits of current prevention and control measures, including PEP policies, with extrapolation across the Philippines.

## Methods

From January 2020 to December 2022, we established a 3-year implementation study of IBCM in Oriental Mindoro Province, Region IV-B, MIMAROPA, Philippines. Here, we utilized a decision tree framework adapted from previous studies^[2,15,16]^ to estimate annual numbers of rabid dogs, human rabies exposures, human rabies deaths/disability-adjusted life years (DALYs) averted, the cost per human death/DALY averted, and the probability of rabies-exposed persons receiving PEP. Within this framework, we used government health and population data, enhanced surveillance data collected through IBCM, and parameter values derived from country-specific data and the literature. Model estimates were used to evaluate current surveillance system performance and analyze the cost-effectiveness of PEP policies for the province, which were extrapolated across the country. Data analysis and figures were undertaken using the R programming language^[17]^. Code and de-identified data to replicate analyses are provided via the GitHub repository: https://github.com/boydorr/OrMin_IBCM_decision_tree.

### Study site and health/agriculture systems

**Figure 1:**
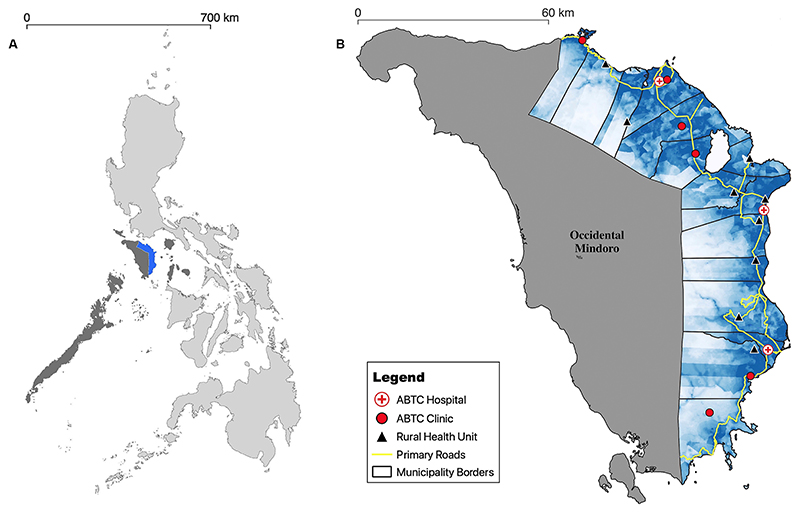
Location of the province of Oriental Mindoro, Philippines. (A) Philippines map showing the MIMAROPA region (dark gray) which includes the province of Oriental Mindoro (blue); (B) Oriental Mindoro Province on the island of Mindoro, showing municipality borders, the human population density (blue), major primary roads (yellow), nine ABTCs: three at major hospitals (white dots with red crosses) and six at community-level clinics (red dots), and nine Rural Health Units (black triangles) that referred bite patients for PEP (i.e., without ABTCs). Human density was calculated at the barangay (village) level from 2020 census data^[18]^. The adjacent province of Occidental Mindoro is labeled and shown in gray. Polygon and line data were sourced from UN-OCHA Humanitarian Data Exchange Project^[19]^. ABTCs: Animal Bite Treatment Centers; PEP: post-exposure prophylaxis.

Canine rabies is endemic throughout Oriental Mindoro [Figure 1], located in the MIMAROPA region which consists of five provinces: Occidental Mindoro, Oriental Mindoro, Marinduque, Romblon, and Palawan. With a human population of 908,339 (2020), the province of Oriental Mindoro comprises 15 municipalities, including the capital city of Calapan^[18]^. At the time of the study, the dog population size and demographics (including ownership status) were unknown. As of 2022, there were nine health facilities with accredited ABTCs administering PEP and nine Rural Health Units (RHU), without ABTCs, providing wound care and referring patients (to ABTCs) for PEP. Of the nine ABTCs, three were major hospitals where most bite patients presented, while the other six were community-level clinics that received fewer patients.

Patients presenting with clinical signs of rabies were not treated at ABTCs but rather admitted to major hospitals for palliative care. ABTCs were financed by their associated hospital/clinic, with PEP jointly financed by the Department of Health (DOH) and an allocated budget from the local government. The advantage and motivation for ABTC accreditation was securing additional funding and the claim to health insurance. This network of ABTCs/RHUs spanning the province acted as a valuable sentinel for collecting enhanced rabies surveillance data through IBCM, with at least one nurse per municipality recruited and trained for the study. The Regional Animal Disease Diagnostic Laboratory (RADDL) for the MIMAROPA region, where most diagnostic testing was completed for this study, is located just south of Calapan.

In Oriental Mindoro, PEP was administered following the updated Thai Red Cross intradermal (ID) regimen (days 0, 3, 7, and 28)^[20]^. The government procures 0.5 mL vials (Speeda, China) that typically provide four 0.1 mL ID doses with wastage of the last 0.1 mL. A complete course, therefore, requires a total of two vaccine vials, considering that each patient should receive two 0.1 mL ID injections per visit. National PEP administration protocols are based upon WHO categories of contact, comprising Category I (non-exposure event from touching or feeding an animal, or licks on intact skin; PEP not indicated); Category II (exposure via nibbling of uncovered skin, minor scratches or abrasions without bleeding: post-exposure vaccination indicated); and Category III (exposure via transdermal bites or scratches, contamination of mucous membranes with saliva from licks, licks on broken skin, exposure due to direct contact with bats: post-exposure vaccination and rabies immunoglobulin indicated)^[1]^.

Protocols in the province generally followed WHO guidance, specifying that equine rabies immunoglobulin (ERIG) be administered primarily to Category III bites on their first presentation. For ERIG, one vial (5 mL EQUIRAB) per patient was provided for free and any additional vials were required to be purchased by the patient. Typically, any contact/bite patient that presented to an ABTC received PEP regardless of their risk of rabies (i.e., including Category I non-exposure events from healthy vaccinated animals). In rare occurrences of PEP stockouts, ABTC nurses used a more risk-based approach to make PEP decisions, saving the free government-supplied PEP for more severe Category II and III events. However, during stockouts, PEP was still available for any person to buy from private health facilities.

Each municipality has a Municipal Agriculture Office (MAO) responsible for managing crops, fisheries, livestock, and, to a lesser extent, domestic animals, such as dogs. MAOs report directly to the mayor’s office. However, for animal health activities at the provincial level, MAOs are often supervised by the Provincial Agriculture Office and the Provincial Veterinary Office (Pro-Vet), which reports to the Bureau of Animal Industry - all of which fall under the jurisdiction of the Department of Agriculture. Most MAOs have animal health staff assigned to livestock, but few or no veterinarians. Veterinary capacity is concentrated near Calapan, where the government employs city vets and there are around five private practices. While these vets could increase the capacity of government-led initiatives, they are currently not mandated to conduct rabies control measures.

The Anti-rabies Act of 2007 enacted responsible pet ownership ordinances, specifying that all dogs be registered, regularly immunized against rabies, not allowed to roam freely, and that bite events be reported within 24 h, with medical expenses shouldered by the animal’s owner. Enforcement of these ordinances and initiation of anti-rabies activities, such as mass dog vaccination (MDV), should be carried out by the MAO if sufficient funds have been allocated via their budget plan. However, many MAOs have insufficient capacity (i.e., trained vaccinators) for MDV and often require additional logistical support and funding from the Pro-Vet. Dog registration, costing owners 20 Philippines pesos (~$0.40 USD), and MDV, offered free to owners, are typically conducted annually from March (Rabies Awareness Month) to June.

Given the decentralized government structure, achieved vaccination coverage heavily relies on the budget allocated by the local government, resulting in notable variation between municipalities. This variability also extends to the MDV strategy, such as house-to-house *vs*. central point, and the protocols employed, including dog age/health restrictions. While some municipalities allocated zero funds for MDV, others allocated upwards of 100,000 Philippines pesos (~$2,000 USD) per year. Due to the COVID-19 pandemic, almost all MDV campaigns were canceled in 2020 and 2021, leading to much lower vaccination coverage. Moreover, the dog population and proportion of roaming dogs were thought to have increased during lockdowns due to more people purchasing pets and reduced animal sterilization.

### Data collection

#### Government surveillance, health, and population data

Human population data from the 2020 government census^[18]^ were utilized to estimate the dog population and denominators for bite patient and rabies exposure incidence in Oriental Mindoro Province. To evaluate surveillance performance, laboratory diagnostic data for animal samples tested for rabies were obtained from RADDL, including both direct fluorescent antibody tests (DFA) and lateral flow devices (LFD). Brain samples were collected by either trained MAO or RADDL staff, adhering to safety and quality protocols (e.g., use of personal protective equipment and transportation in cold boxes). In cases where RADDL was unable to complete DFA diagnostic testing (e.g., due to lack of a working fluorescent microscope or a broken storage freezer), LFD testing was conducted at RADDL, and then samples were sent to the Research Institute for Tropical Medicine (RITM) in Manila for confirmatory DFA. All diagnostic test results for samples from Oriental Mindoro were consolidated in RADDL records.

To summarize bite patient characteristics, we relied on Provincial Health Office (PHO) quarterly and annual reports compiled from ABTC patient logbooks. Initially recorded on paper, these records were later entered electronically at the end of each month. Collected for the National Rabies Information System (NaRIS) since 2007, these records included patient details such as demographics, wound location, WHO category of contact, species of biting animal, and information about PEP administration and compliance^[21]^. These data were used to estimate the incidence of bite patient presentations for prospective comparison with IBCM records to determine the completeness of risk assessments performed (numerator) over total bite patients visiting ABTCs (denominator), and for subsequent extrapolation. We used reports from investigations of human deaths by the PHO to summarize human rabies cases from 2020-2022.

Lastly, we utilized PHO budget/procurement reports to gather data inputs related to PEP costs, including the average number of doses received per bite patient, as well as the frequency of ERIG usage. For estimating PEP costs, we considered both human rabies vaccine and ERIG expenses. However, the estimates used to evaluate cost-effectiveness did not include PEP administration costs (e.g., personnel, syringes, *etc*.), as these were covered by the health system budget. When extrapolating estimates across the Philippines, we referred to NRPCP/DOH records to determine the average number of bite patient presentations and human rabies deaths reported nationally^[8]^.

#### IBCM data

IBCM data were collected over 3 years (January 2020 to December 2022). ABTC nurses received training to perform risk assessments for bite patients, while animal health workers from the MAO were trained to investigate suspect animals. In addition to data required for NaRIS, we requested nurses to record information about the biting animal (e.g., health, vaccination, and ownership status) and circumstances of the bite event. Similarly, animal health workers were tasked with collecting initial and follow-up data on the biting animal throughout the observation period, requesting Pro-Vet support to euthanize if necessary, collecting, storing, and transporting samples to RADDL, and conducting LFD testing in the field. Risk assessment and animal investigation data were submitted via standardized forms through a bespoke mobile phone-based application adapted for the Philippines^[22]^. IBCM data for this study were not integrated into NRPCP records.

IBCM protocols specified that nurses trigger an investigation by contacting their designated animal health counterpart at the MAO if the biting animal was suspected of rabies. Animal health workers would then conduct the animal investigation either via phone or in person. If the animal was found alive and healthy, MAO staff would follow up with the animal owners over the 14-day observation period, as specified by NRPCP guidelines (Administrative Order No. 2018-0013)^[7]^. In cases where the animal displayed signs of rabies, protocols dictated that the Pro-Vet should assist in euthanizing the animal, and a sample should be collected to test for rabies. However, animals had often died or were killed by the owners/community prior to the investigation.

If the animal died or was euthanized, a brain sample was collected by trained MAO or RADDL staff for diagnostic testing, usually within one day. LFDs (BioNote, Inc, Hwaseong, Korea) were provided to animal health workers and RADDL staff for in-field and laboratory-based testing. Findings from a study in the Philippines (184 samples) reported Bionote LFD sensitivity of 0.95 and specificity of 1.00 compared to DFA results^[23]^. For the majority of cases, the MAO staff brought the animal’s head or carcass to RADDL for testing. Animal cases were confirmed either at RADDL or RITM with DFA, as recommended by WHO^[24]^. All samples collected during the study that tested positive by LFD were also confirmed positive subsequently by DFA, but not all samples that were tested by DFA were also tested by LFD.

Patient risk categories were not updated from animal investigations, which could not be consistently collected due to COVID-19 pandemic restrictions. Thus, for this study, patients were classified as either low-risk, unknown-risk, or high-risk for rabies exposure based on the patient risk assessment from their first visit to the ABTC, apart from laboratory-confirmed biting animals, for whom risk categories were updated retrospectively. The risk of exposure categories used in this study was based on WHO animal case definitions^[1]^, where at least one criterion was met:

**Low-risk:** (WHO definition “Not a case”) Biting animal had no clinical signs of rabies and was healthy and alive 14 days after the bite/exposure event or tested negative for rabies (if euthanized/killed).
**Unknown-risk:** (WHO definition “suspected” or “probable”) Biting animal not identified or found; therefore, the history of the animal was unknown (e.g., vaccination/health status, contact with suspected, probable, or confirmed rabid animal, health status, *etc*.).
**High-risk:** (WHO definition “suspected”, “probable” or “confirmed” animal case) Biting animal showed clinical signs of rabies (e.g., aggressive/erratic behavior, hypersalivation, paralysis, tremors, abnormal vocalization, loss of appetite); had a history of contact with suspect/confirmed rabid animal; and died within 14 days of exposure event; or tested positive for rabies.

### Data analysis

#### Decision tree model

We used a decision tree framework to probabilistically describe the steps by which rabies infection in dogs leads to human exposures and deaths, and associated costs. This type of framework has been used before to estimate the burden of rabies^[15,16]^. Here, we extended the framework using IBCM data and further estimated current surveillance performance and cost-effectiveness of prevention measures.

To simplify our analysis, we made several assumptions. We assumed that all bite patients who reported to an ABTC received PEP (and that PEP was 100% effective in preventing rabies), considering that shortages and vaccine refusal are rare in Oriental Mindoro. Additionally, we assumed that reported human rabies deaths were recorded correctly with high probability (P_obs|death_). Our estimates and 95% prediction intervals (PrI) were based on 1,000 probabilistic draws of parameters described in Table 1, following the decision tree framework.

IBCM risk assessment classifications were used to assign patients as either bitten by healthy dogs (low-risk) or rabid dogs (high-risk), with uncertainty based on the observed range in IBCM risk assessments (lower limits included only high-risk, while upper limits included high-risk plus unknown-risk). We used the proportions of high-risk bites from both incomplete IBCM data and complete data from one ABTC to extrapolate to the province. These estimates were compared to each other and resulting estimates from the decision tree model.

**Figure 2:**
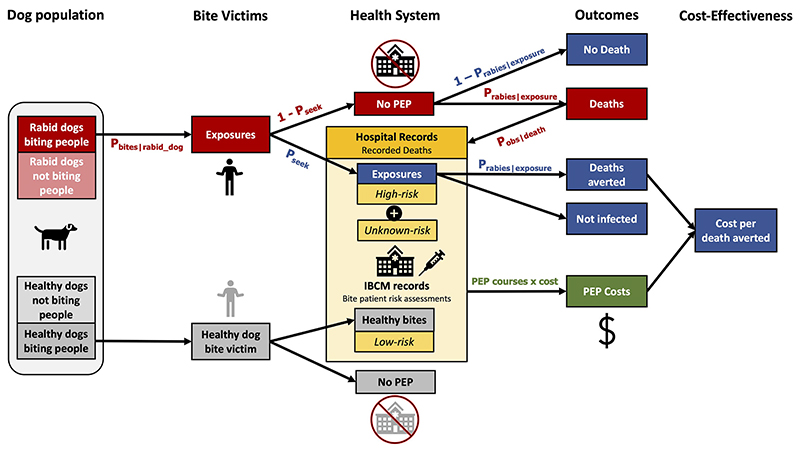
Schematic of decision tree used to estimate the burden of rabies, deaths averted by PEP, and associated costs. This framework illustrates the steps taken to probabilistically estimate outcomes associated with rabies infections in dogs and resulting human exposures and deaths. IBCM and hospital record (PHO) data inputs are shown in yellow boxes. Rabies exposures that can lead to rabies deaths in the absence of PEP are shown in red boxes; rabies exposures where risk is mitigated via PEP are shown in blue boxes; and healthy dog bites are shown in gray boxes. IBCM: Integrated Bite Case Management; PEP: post-exposure prophylaxis; PHO: Provincial Health Office.

Total exposures were calculated as the sum of high-risk exposures, assigned prospectively, that sought PEP (from IBCM data) and estimated exposures that did not seek PEP extrapolated from recorded human rabies deaths. Similarly, numbers of rabid dogs were estimated from total exposures and the average number of people bitten per rabid dog, P_bites|rabid_dog_. Details of calculations are described below and outlined in Figure 2.

#### Parameter estimates

Parameters for the model used government or IBCM data from the Philippines to reflect local context where possible [Tables 1 and 2]. When national or regionally specific data were not available, we used probabilities from the literature, including P_rabies|exposure_ and P_bites|rabid_dog_ calculated from contact tracing data from Tanzania^[25,26]^.

We estimated the probability of rabies-exposed bite victims seeking PEP (P_seekPEP_) from the probability of developing rabies following exposure in the absence of PEP (P_rabies|exposure_), IBCM risk assessments, and observed rabies deaths (D_observed_). We assumed deaths were observed with high probability (P_obs|death_): D_Total_ = D_observed_/P_obs|death_.

To estimate total exposures (E_Total_), we summed estimates of exposures who did not seek PEP (E_no_PEP_) with exposures who did (E_PEP_) derived from the IBCM risk assessments:

$$\begin{array}{l} {\text{E}}_{\text{no\_PEP}}={\text{D}}_{\text{Total}}/{\text{P}}_{\text{rabies|exposure}}\\ {\text{E}}_{\text{Total}}={\text{E}}_{\text{no\_PEP}}+{\text{E}}_{\text{PEP}} \end{array}$$

P_seekPEP_ was calculated from the estimates of total exposures and exposures that did not seek PEP (P_seek_ = E_no_PEP_/E_Total_), while deaths averted (D_averted_) were estimated from rabies-exposed patients who sought PEP and the probability of developing rabies following exposure in the absence of PEP: D_averted_ = E_PEP_ × P_rabies|exposure_.

The dog population was estimated from the human population as recorded in the national census^[18]^, divided by the human:dog ratio (HDR). A range of HDRs were extracted from published studies in the Philippines^[27,28]^. Rabid dogs were estimated from total exposures (E_Total_) divided by P_bites|rabid_dog_ and annual rabies incidence per 1,000 dogs calculated. The percentage of rabid dogs that were laboratory-confirmed out of the estimated total rabid dogs was calculated from RADDL data.

Annual PEP costs were calculated from the patients who received post-exposure vaccine multiplied by the cost of the vaccine course, and patients who received ERIG multiplied by the average cost of ERIG, with cost variables described in Table 2. The average cost per death averted was estimated as total PEP costs divided by estimated deaths averted. Similarly, the average cost per DALY averted was estimated by dividing total PEP costs by DALYs averted.

DALYs were calculated from Years of Life Lost (YLL). For rabies, Years of Life lived with Disability are considered insignificant due to the acute and fatal nature of rabies, and therefore were not included in DALY estimates. To estimate YLL, we used expected global life expectancy^[29]^ and subtracted the mean age of deaths recorded in Oriental Mindoro during the 3-year study period.

#### Sensitivity analysis

We conducted sensitivity analyses comparing model estimates of human deaths, total exposures, P_seekPEP_, rabid dogs, and deaths averted across a range of uncertainty to examine the influence of parameter values. For our probabilistic sensitivity analysis, we took 1,000 random draws across the specified distributions in Table 1 for HDR, E_PEP_, P_obs|death_, P_rabies|exposure_ and P_bites|rabid_dog_ (including uniform, binomial and negative binomial) and across a uniform distribution for the range of human deaths and confirmed cases over the three years. Some variables (e.g., HDR) that had high uncertainty in the baseline analysis remained unchanged for the sensitivity analyses.

### Ethics statement

Ethical approval was obtained from the Research Institute for Tropical Medicine (RITM), Department of Health (2019-023), and the University of Glasgow College of Medical, Veterinary & Life Sciences (200190123).

## Results

### Characteristics of bite patients presenting to ABTCs

Between January 2020 and December 2022, a total of 33,947 bite patients presented to ABTCs in Oriental Mindoro to receive PEP for animal contact or bite events. This equates to an average of 11,316 (min = 8,370, max = 14,308) bite patients per year, 943 per month (min = 698, max = 1,192), and an annual incidence of 1,246 bite patient presentations per 100,000 people over the study period. Characteristics of bite patients recorded in ABTC logbooks and then documented in PHO records are described in Table 3, including data from the year prior (2019) to the implementation of IBCM for comparison before the COVID-19 pandemic.

Of the biting animals reported through patient presentations to ABTCs, 67.8% were dogs (23,004/33,947) and 31.5% were cats (10,693/33,947), with < 1% from other species (250/33,947). An average of 42.2% of bite patients were under the age of 15 years, which is higher than this age group proportion in the general population (32.03%)^[18]^, demonstrating a greater risk of rabies exposure for children. Most bite victims that presented to health facilities over the study were Category II (79.5%) and Category III (19.5%), with only 1.1% being Category I non-exposure events. Of the Category III patients that presented to ABTCs, 79.6% received ERIG (15.5% of total bite patients) on their first visit, following PEP guidelines mandated by the DOH.

### Risk of rabies exposure and human deaths

**Figure 3:**
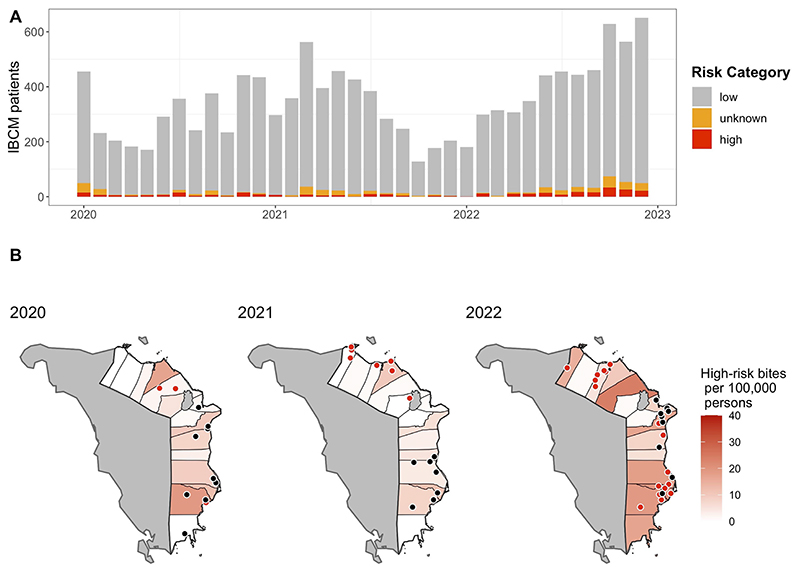
IBCM risk assessment and rabies case data from Oriental Mindoro Province. (A) Time series from January 2020 to December 2022, showing IBCM bite patient data by risk category: low-risk (gray), unknown-risk (orange), and high-risk (red); (B) Maps showing the incidence of high-risk bites per 100,000 persons from IBCM risk assessments by municipality (red shading) and locations where exposure events occurred for human rabies cases (black dots) and where confirmed animal cases were found (red dots) by year: 2020, 2021, and 2022. IBCM: Integrated Bite Case Management.

Due to high patient volumes, busy workloads, and duplication with government reporting systems (i.e., NaRIS), risk assessments were not collected for all patients presenting to ABTCs as initially planned in study protocols. IBCM data were collected for 37.2% of total PHO-recorded bite patients, corresponding to 12,640 records: 3,623 in 2020, 3,924 in 2021, and 5,093 in 2022. Of the IBCM patient records, 2.5% (312/12,640) were assessed to be high-risk (5.7% for high-risk + unknown-risk, 715/12,640) for rabies exposure (Figure 3A, i.e., biting animals were considered “probable” or “confirmed” rabies by WHO case definitions). Of the 312 classified as high-risk bites, 240 (76.9%) were from dogs and 72 (23.1%) from cats, with most being WHO Category II (64.7%) and Category III (34.6%). At the time of the risk assessment, 259 (83%) of the high-risk biting animals had died or been killed/euthanized, and 89 (28.5%) were assessed as suspicious for rabies by the nurse based on the bite patient’s description of the animal’s history, while an additional 27 (8.7%) were assessed as “sick, not rabies”.

Extrapolating the proportion of high-risk bites from IBCM data (2.5%) to total bite patients in the province (33,947), we estimate 838 high-risk bites over 3 years, with an average of 279 per year. One ABTC, located in a major hospital in Calapan, reported nearly complete data during the study. These data represented 47.9% (6,055/12,640) of IBCM records, with 0.96% of bites assessed to be high-risk (3.7% for high-risk + unknown-risk). Using these proportions for comparison with incomplete IBCM data, we estimated 325 high-risk bites over 3 years and an average of 108 per year. When assuming only dog bites are high-risk, based on RADDL records which found no cats tested positive over the last 5 years, we estimate 2.8% (240/8,701) of bites to be high-risk (5.2% for high-risk + unknown-risk, 449/8,701).

Over the course of the 3 years (2020-2022), 25 human deaths were formally investigated and recorded as probable rabies cases in Oriental Mindoro Province, and 28 animal cases were confirmed with DFA. Death investigations, conducted by a team of PHO and/or DOH staff, involved clinical diagnosis using hospital records and interviews with medical staff and the patient’s family. No samples were collected for testing. Deaths ranged in age from 4 to 69 years (median = 37 years), with 6 (25%) being under 15 years old and a male:female ratio of 1.08:1. Exposure events leading to human infection then death were concentrated in 8 of the 15 municipalities [Figure 3B], with 64% occurring in just three municipalities (Bongabong-6, Mansalay-5, and Pinamalayan-5). The most densely populated area, the capital city of Calapan, had zero human rabies deaths but had two animal cases confirmed over the study period.

For all reported human rabies cases, the biting animal was a dog. Of the confirmed animal cases, 54% (15/28) were found in three municipalities (Baco-7, Mansalay-4, and Puerto Galera-4), of which two (Baco and Puerto Galera) reported zero human rabies deaths during the study period. None of the human cases received PEP prior to displaying symptoms of rabies infection. As per PHO death investigation reports, primary reasons for not seeking PEP after exposure events included: a lack of awareness of the risk of rabies from animal bites, the choice to consult traditional healers for treatment (known as tandok/tawak in the Philippines), and financial constraints preventing the ability to cover travel costs and take time off work to seek PEP, particularly for those in remote locations relying on agricultural work.

### Decision tree estimates of rabies burden and surveillance performance

From the decision tree model [Figure 2], we estimated that an average of 216 people (95% PrI 91-408) were exposed to rabies annually in Oriental Mindoro [Table 4], with an average of 55 (95% PrI 42-79) not reporting to health facilities for PEP, i.e., people exposed to rabies sought PEP with probability 0.738 (95% PrI 0.594-0.811), assuming 90% of rabies deaths are recorded (i.e., P_obs|death_ = 0.9). Under this same assumption, around 27 (95% PrI 25-35) human deaths were estimated to have occurred over the 3 years (in comparison to the 25 deaths recorded). While the PHO records [Table 3] indicate a high incidence exceeding 1,240 bite patient presentations per 100,000 people per year, we estimated an annual incidence of 24 (95% PrI 10-45) exposures and 0.77-1.1 deaths per 100,000 people [Table 4].

We estimated there were an average of 560 (95% PrI 217-1,090) rabid dogs per year in Oriental Mindoro, from an estimated dog population of 140,420 (95% PrI 92,340-289,950), equating to 3-5 rabid dogs per 1,000 dogs/year. These estimates suggest that surveillance only detected between 1%-2% of animal cases during the study period. Though low, animal surveillance performance in terms of laboratory-confirmed cases increased almost fourfold from 2020 to 2022 (from 0.59% to 2.3%) through implementing IBCM. However, this increase in case detection may also indicate higher incidence in 2022 compared to 2020, rather than improved surveillance performance.

Decision tree estimates revealed considerable variation in rabies burden and surveillance performance by municipality [Table 5]. The estimated exposure incidence ranged from 4 to 59 people per 100,000 who were potentially exposed to rabies each year across the 15 municipalities. Animal surveillance was weak, with the number of recorded human deaths (25 total) over 3 years nearly matching the number of confirmed animal cases (28 total). In 12 of the 15 municipalities, < 2% of estimated animal cases were detected, with four municipalities not submitting any samples for diagnostic testing. Notably, the two municipalities with the highest animal case detection, Baco (13.7%) and Puerto Galera (4.1%), did not record any human rabies deaths.

**Figure 4:**
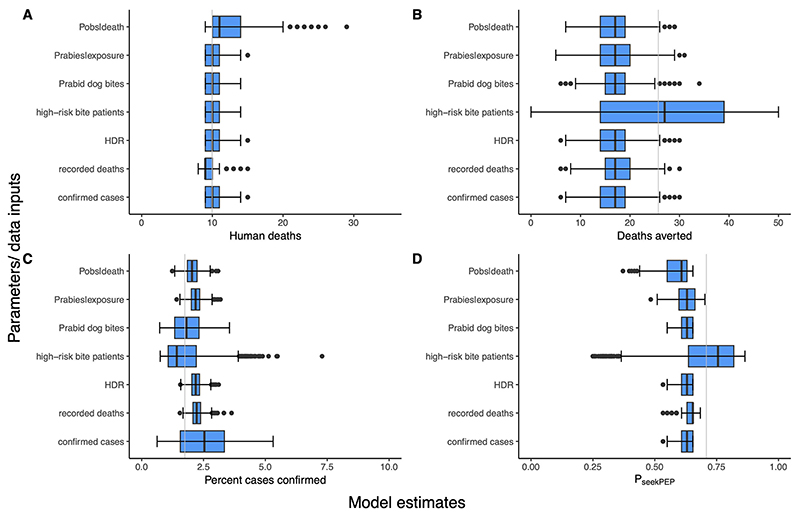
Model sensitivity to uncertainty. Variation in model estimates (x-axis) of (A) annual human rabies deaths; (B) human rabies deaths averted; (C) the percentage of rabid animals confirmed; and (D) probability of rabies exposures obtaining PEP. Model parameters [Table 1] and data inputs that were varied in the sensitivity analysis are shown on the y-axis. Variations in estimates are not symmetrical around the baseline estimate (vertical gray line) because the range of uncertainty examined was not symmetric distributions centered on the baseline parameters. HDR: Human:dog ratio; PEP: post-exposure prophylaxis.

From the sensitivity analysis [Figure 4], the parameters that had the greatest impact on estimates of human rabies exposures and P_seekPEP_ were the number of high-risk bites, followed by the probability of observing human deaths (P_obs|death_). The probability of a rabid dog biting (P_bites|rabid_dog_) and the number of high-risk bite patients most influenced estimates of rabid dogs.

### Economic analysis of PEP policies and costs

We calculated an average PEP cost of $51.44 USD per person ($37.50 USD for those receiving vaccine only, and $127.50 USD for those also receiving ERIG), based on the assumption that each patient received an average of six 0.1 mL ID injections of post-exposure vaccine, and that 79.6% of Category III bites (15.5% of total bite patients) received ERIG, with an average of 2 vials of ERIG each. This translates to total costs (human rabies vaccine and ERIG) ranging between $445,185 and $734,280 USD annually and over $1.74 million USD during the 3-year study period (2020-2022) in Oriental Mindoro.

We estimated that PEP prevented between 20 and 43 deaths (95% PrI 3-72) per year in Oriental Mindoro at an average cost of $20,190 USD (95% PrI $11,565-79,400) per death averted. Using the mean age of death during our study period (35 years), we estimated an average of 1,105 DALYs averted annually, costing $527 USD per DALY averted. If PEP were administered solely to high-risk and unknown-risk bite patients during the 3 years (715 total), estimated costs would be approximately $17,050 USD annually for vaccine (~$11,920 USD) and ERIG (~$5,130 USD), assuming a full vaccine course (8-ID injections) and all Category III bites received ERIG. By providing PEP only to bite patients with exposure risk, estimated costs would decrease to $591 USD per death averted.

Upon extrapolating these findings nationwide using NRPCP bite records (> 1.1 million bite patients presenting to ABTCs annually), we project expenditures surpassing $56.6 million USD on human rabies vaccine (> $41.2 million) and ERIG (> $15.3 million, assuming 15.5% of bite patients receive ERIG) each year. Assuming 2%-3% of bites presenting to ABTCs are probable rabies exposures and utilizing DOH national records reporting 200-300 deaths/year, we estimate that PEP prevents roughly 3,520 to 5,570 deaths each year in the Philippines, at an average cost of $12,460 and $325 USD per death/DALY averted, respectively. However, these estimations are conservative, considering increasing PEP-seeking behaviors and the likelihood of underreported human deaths.

## Discussion

### Key findings

The findings from our analysis reveal that despite an overall bite patient incidence exceeding 1,240/100,000 persons per year, the majority (> 97%) of patients who sought PEP in Oriental Mindoro had encountered non-exposures from healthy animals. The Philippines’ national policy mandating free PEP provision and widespread establishment of ABTCs has substantially improved PEP access, preventing an estimated average of 29 deaths annually throughout the province. Nevertheless, even with increased availability and accessibility, only around 73.8% of people exposed to rabies were estimated to seek PEP provincewide. Consequently, dog-mediated rabies still precipitated 7 to 9 reported human deaths (0.77 to 0.99 per 100,000 persons/year) in Oriental Mindoro Province each year of the study.

The distribution of the human rabies burden was not uniform across the province, as evidenced by three municipalities accounting for 16 out of 25 deaths over the 3-year duration of the study. This spatial distribution of human cases likely arose from a combination of factors, including localized outbreaks, inadequate dog vaccination coverage, suboptimal PEP-seeking behaviors, and potential variations in surveillance and case detection capabilities. Mapping the locations of human cases alongside laboratory-confirmed animal cases [Figure 4] clearly illustrated that nearly all animal rabies testing was conducted in northern Oriental Mindoro in 2020 and 2021, whereas reported incidents of human deaths were limited to the central and southern areas of the province. In 2022, sample collection increased throughout the province; however, reported human cases remained in central and southern municipalities. The incidence of high-risk bites showed a notable increase in 2022, likely due to the cancellation of MDV campaigns in 2020/2021 and the lifting of COVID-19 movement/travel restrictions, potentially leading to more exposure events.

Our findings indicate that although human case detection is relatively robust, animal surveillance should be enhanced to capture the incidence of rabies more effectively within the dog population. Over the 3-year study, our decision tree model estimated a total of 1,678 rabid dogs (95% PrI 1,016-2,386) may have been present in Oriental Mindoro. Yet only 28 animal cases were laboratory-confirmed during this time (case detection of 1.7%). Notably, three municipalities accounted for more than half of positive dog cases (15 of 28), indicating stronger surveillance, though not necessarily a higher incidence of dog rabies. IBCM surveillance protocols, which encouraged the investigation of suspected rabid animals and the collection of samples in the case of dead or euthanized animals, led to a nearly fourfold increase in the detection of laboratory-confirmed dog rabies cases from 2020 to 2022. However, external factors such as the COVID-19 pandemic and minimal to no dog vaccination in 2020 and 2021 make it difficult to discern whether the higher case detection was exclusively due to surveillance being enhanced by IBCM or because of increased rabies incidence within the dog population.

### Strengths and limitations

We were typically able to classify biting animals as broadly “high-risk” or “low-risk” using initial patient risk assessments from IBCM, but these did not always provide adequate information to differentiate between WHO classifications “suspect” or “probable”. IBCM protocols specified risk assessments for every bite patient presenting to ABTCs and investigations of any animal deemed high-risk. However, the COVID-19 pandemic and ensuing lockdowns contributed to challenges in the delivery of IBCM training and subsequent implementation of protocols. Heavy workloads and temporary closure/reduced operating hours of ABTCs limited the capacity of health workers to complete/submit risk assessments, while movement restrictions prevented in-person animal investigations and affected sample collection. Challenges associated with COVID-19 primarily affected IBCM implementation in 2020 and 2021, with 2022 mostly returning to a relatively normal situation.

There was a potential bias towards the submission of high-risk bite data due to higher prioritization of reporting, which may have resulted in overestimating rabies exposure incidence. However, attempts were made to adjust for this by using nearly complete risk assessment data from one ABTC, located in the capital city of Calapan, as well as the incomplete IBCM data submitted from all ABTCs, to extrapolate to the province. Assuming there are differences in PEP-seeking behavior and endemicity of dog rabies between urban and rural settings, both estimates come with limitations. However, these two methods of extrapolation provide comparisons for our decision tree estimates and further evidence that only a small percentage (< 3%) of bite patients seeking PEP were likely true rabies exposures.

Additional limitations include simplifying assumptions and uncertainties in our decision tree model parameters. The parameters describing rabid dog biting (P_bites|rabid_dog_) and the probability of infection following exposure (P_rabies|exposure_) were from a different context (Tanzania), potentially limiting the accuracy of results specific to the Philippines. While the probability of infection following exposure (P_rabies|exposure_) likely has minimal variation between contexts, the probability that a rabid dog will bite (P_bites|rabid_dog_) may be context-specific due to differences in factors such as the dynamics of animal/human behaviors within the community, cultural norms (e.g., whether dogs are allowed to roam), and the density of human and dog populations. Further research estimating these parameters specific to the Philippines would be useful for future studies.

Uncertainty in P_rabies|exposure_ had little impact on model estimates; however, P_bites|rabid_dog_ affected estimates of rabid dogs, and lower assumptions of P_obs|death_ led to estimates of P_seekPEP_ deemed implausibly low for the province. We consider it reasonable to assume that most human rabies deaths in the Philippines are reported and captured in provincial and national statistics in contrast to some other contexts, for example, in Sub-Saharan Africa, where much fewer deaths are reported. This means that the P_obs|death_ parameters used in this model are specific to the Philippines and would require adjustment when applied to other countries or regions.

### Wider context

Our results from Oriental Mindoro were comparable to findings from other IBCM case studies in the Philippines. A high incidence of bite patients presenting to ABTCs was found in the provinces of Bohol in 2013 (> 300/100,000 persons per year) and Albay in 2018-2019 (> 600/100,000 persons per year), with most bitten by healthy animals (> 92% in Bohol and > 97% in Albay)^[12,13]^. Similar to our estimates from Oriental Mindoro Province (24 per 100,000 persons per year), these data roughly translate into an estimated incidence of rabies exposures of 24 (Bohol) and 18 (Albay) per 100,000 persons per year. This consistency in findings indicates that while PEP-seeking behaviors have increased unsustainably in the Philippines since the initiation of the free PEP policy in 2007, the average risk of rabies exposure has remained relatively consistent across much of the country. Moreover, over the last decade, the number of human rabies cases reported has continued to fluctuate between 200 to 300 deaths per year, despite the continuous expansion of ABTC infrastructure and increased expenditure on and access to free government-supplied PEP^[7,8]^.

### Conclusions and recommendations

The NRPCP has executed a comprehensive package of rabies control measures, engaging community and intersectoral involvement from the national to local level and vastly expanding PEP accessibility. Even so, the current animal surveillance system does not sufficiently capture the burden of rabies in the dog population, and dog vaccination coverage remains inadequate. While government-allocated budgets for rabies control continually shift with different administrations, the human health sector typically receives funding upwards of tenfold higher than the animal health sector. To achieve rabies elimination, emphasis must be placed on developing effective strategies and funding dog vaccination to reduce the incidence of rabies in the reservoir dog population. Although free PEP policies are important to ensure the accessibility of these emergency measures, they will not eliminate rabies or reduce the risk of exposure.

Our study suggests that for improved access to PEP to remain cost-effective, it should be implemented in conjunction with strengthened rabies surveillance that provides more accurate data on the risk of exposure for bite patients^[3]^. Using a risk-based approach to inform PEP decisions has the potential to reduce unnecessary spending on PEP for events that pose no risk of rabies exposure. However, switching to more judicious PEP provisioning is likely to be difficult in the Philippines given current established practices. Considerable training and local buy-in would be needed to ensure that health workers are confident and supported in their decision-making and, more critically, that the risk of rabies exposure is reduced and ideally eliminated through mass dog vaccination.

In conclusion, our findings demonstrate the wider benefits of integrating IBCM into national policy in the Philippines. If implemented effectively, IBCM has the potential to guide judicious PEP administration, thereby improving cost-effectiveness and allowing the reallocation of funds to the animal health sector for dog vaccination - the most effective way to eliminate rabies. Moreover, IBCM can provide more accurate data on the circulation of rabies to inform control through mass dog vaccination and help achieve and maintain rabies elimination^[30]^.

## Figures and Tables

**Table 1:** Parameters and data used in the decision tree model

Definition	Parameter	Distribution/Data	Point estimate/range for main model	Range used for sensitivity analyses	Source
Probability of developing rabies after exposure in the absence of PEP	P_rabies|exposure_	Binomial	0.165	0.124-0.206	[25]
Average number of persons bitten per rabid dog	P_bites|rabid_dog_	Negative binomial	*μ* = 0.3862 *k* = 0.7055	*μ* = 0.15-0.50 *k* = 0.7055	[26] Ferguson, E. pers. comm.
Probability of human rabies death being recorded in official records	P_obs|death_	Binomial	0.90	0.5-1.0	Assumption, based on discussion with stakeholders
Rabies exposures receiving PEP	E_PEP_	IBCM data	High-risk bite patients - (high-risk + unknown-risk)	high-risk dog bite patients/2 - (high-risk + unknown risk bite patients)	IBCM data (see text for details)
Human:dog ratio for Oriental Mindoro	HDR	Uniform	3-10	3-10	[27,28]

HDR: Human:dog ratio; IBCM: Integrated Bite Case Management; PEP: post-exposure prophylaxis.

**Table 2:** Cost variables relating to PEP provisioning

Definition	PEP cost variables	Source
Average number of vaccine vials per patient (0.5 mL Speeda)	~2	Oriental Mindoro PHO bite patient, budget, and financial records^[13]^
Average cost of 1 vaccine vial (0.5 mL Speeda)	$25 USD	
Average number of ID injections per patient	~6	
Cost of 1 ID injection of vaccine	$6.25 USD	
Average number of ERIG vials per patient	~2	
Cost of vial of ERIG (5 mL EQUIRAB)	$45 USD	
ERIG dosage per 5 mL vial	1 vial/25 kg	

ERIG: Equine rabies immunoglobulin; ID: intradermal; PEP: post-exposure prophylaxis; PHO: Provincial Health Office.

**Table 3:** Characteristics of bite patients and human deaths from PHO records before the study (2019) and during the study period (2020 to 2022)

Year	2019 pre-study	2020	2021	2022	Average per study year (2020-2022)
**Recorded human deaths**	5	9	7	9	8
**Total bite patients**	9,217	8,370	11,269	14,308	11,316
**Mean monthly patients**	768	698	939	1,192	943
**Bite incidence per 100 k**	1,015	921	1,241	1,575	1,246
**% male**	49.5	49.9	48.2	47.6	48.4
**Bites U15yrs (%)**	3,781 (41)	3,548 (42.4)	5,065 (44.9)	5,714 (39.9)	4,776 (42.2)
**Category I (%)**	106 (1.2)	307 (3.7)	26 (0.2)	30 (0.2)	121 (1.1)
**Category II (%)**	7,322 (79.4)	6,257 (74.8)	9,189 (81.5)	11,535 (80.6)	8,994 (79.5)
**Category III (%)**	1,789 (19.4)	1,806 (21.6)	2,054 (18.2)	2,743 (19.2)	2,201 (19.5)
**ERIG (% of Category III)**	1,445 (80.8)	1,459 (80.8)	1,603 (78)	2,197 (80.1)	1,753 (79.6)
**Dog bite (%)**	6,311 (68.5)	5,947 (71.1)	7,768 (68.9)	9,289 (64.9)	7,668 (67.8)
**Cat bite (%)**	2,744 (29.8)	2,352 (28.1)	3,429 (30.4)	4,912 (34.3)	3,564 (31.5)
**Bite by other animal (%)**	162 (1.8)	71 (0.8)	72 (0.6)	107 (0.7)	83 (0.7)

ERIG: Equine rabies immunoglobulin; PHO: Provincial Health Office.

**Table 4:** Decision tree model estimates and recorded data for the annual burden of rabies in Oriental Mindoro Province

Year	2020	2021	2022
**Recorded deaths (PHO)**	9	7	9
**Estimated deaths**	10 [9-12]	7 [7-10]	10 [9-13]
**Estimated exposures**	198 [133-258]	149 [91-210]	302 [192-408]
**Estimated exposures not given PEP**	61 [55-73]	42 [42-61]	61 [55-79]
**Estimated exposure incidence per 100,000**	22 [15-28]	16 [10-23]	33 [21-45]
**Laboratory-confirmed animal cases (RADDL)**	3	7	18
**Estimated rabid dogs**	510 [322-715]	388 [217-581]	781 [477-1,090]
**Estimated % confirmed animal cases**	0.59% [0.42-0.93]	1.81% [1.2-3.23]	2.3% [1.65-3.77]
**Estimated rabid dogs per 1,000 dogs**	3.46 [1.55-6.59]	2.57 [1.08-5.43]	5.3 [2.27-10.27]

Median values are shown together with 95% prediction intervals in brackets. Recorded human deaths are from the PHO and animal case data from the RADDL. PEP: Post-exposure prophylaxis; PHO: Provincial Health Office; RADDL: Regional Animal Disease Diagnostic Laboratory.

**Table 5:** Decision tree estimates for the burden of rabies from January 2020 to December 2022 by municipality

Municipality	Population (2020 census)	Estimated dog population	Estimated rabid dogs	Confirmed animal cases	Estimated % confirmed cases	Estimated exposures	Recorded human deaths	Estimated human deaths	Estimated exposure incidence 100 k/year
Baco	39,817	6,140 [4,050-12,520]	51 [14-98]	7	13.73% [7.14%-50%]	19 [8-30]	0	0 [0-0]	16 [6-25]
Bansud	42,671	6,590 [4,390-13,340]	59 [27-103]	0	0% [0%-0%]	22 [15-33]	1	1 [1-2]	17 [12-26]
Bongabong	76,973	12,010 [7,810-24,010]	197 [139-272]	2	1.02% [0.74%-1.44%]	75 [65-91]	6	6 [6-9]	33 [28-39]
Bulalacao	44,366	6,960 [4,510-14,100]	64 [35-106]	0	0% [0%-0%]	24 [20-32]	1	1 [1-2]	18 [15-24]
Calapan	145,786	22,710 [14,920-45,680]	200 [61-366]	2	1% [0.55%-3.28%]	77 [26-131]	0	0 [0-0]	18 [6-30]
Gloria	50,496	7,650 [5,130-15,720]	91 [50-141]	1	1.1% [0.71%-2%]	35 [26-47]	2	2 [2-4]	23 [17-31]
Mansalay	59,114	9,070 [6,000-18,700]	267 [167-382]	4	1.5% [1.05%-2.4%]	104 [70-135]	5	5 [5-8]	59 [40-76]
Naujan	109,587	17,050 [11,180-34,140]	129 [44-229]	2	1.55% [0.87%-4.55%]	50 [19-78]	0	0 [0-0]	15 [6-24]
Pinamalayan	90,383	13,850 [9,220-28,590]	200 [134-288]	1	0.5% [0.35%-0.75%]	78 [58-100]	5	5 [5-8]	29 [21-37]
Pola	35,455	5,620 [3,620-11,230]	105 [62-165]	1	0.95% [0.61%-1.61%]	40 [30-56]	4	4 [4-6]	38 [28-52]
Puerto Galera	41,961	6,560 [4,320-12,950]	98 [27-187]	4	4.08% [2.14%-14.81%]	38 [13-63]	0	0 [0-0]	30 [10-50]
Roxas	58,849	9,250 [5,980-18,530]	158 [97-228]	3	1.9% [1.32%-3.09%]	60 [46-74]	1	1 [1-2]	34 [26-42]
San Teodoro	19,121	3,000 [1,9506,120]	24 [3-62]	0	0% [0%-0%]	9 [2-18]	0	0 [0-0]	16 [3-31]
Socorro	41,585	6,430 [4,200-13,230]	12 [2-37]	0	0% [0%-0%]	4 [1-8]	0	0 [0-0]	4 [1-6]
Victoria	52,175	8,100 [5,280-16,290]	20 [6-47]	1	5% [2.13%-16.75%]	8 [4-11]	0	0 [0-0]	5 [3-7]
Oriental Mindoro	908,339	140,990	1,678	28	Avg = 1.67%	646	25	35	Avg = 24

Median values are in bold and 95% prediction intervals are shown in brackets. Human data is from the PHO and animal data is from the RADDL. PHO: Provincial Health Office; RADDL: Regional Animal Disease Diagnostic Laboratory.

## Data Availability

De-identified data and R programming code to replicate analyses are provided via the GitHub repository: https://github.com/boydorr/OrMin_IBCM_decision_tree.
